# Novel Hit Compounds as Putative Antifungals: The Case of *Aspergillus fumigatus*

**DOI:** 10.3390/molecules24213853

**Published:** 2019-10-25

**Authors:** Eftichia Kritsi, Minos-Timotheos Matsoukas, Constantinos Potamitis, Anastasia Detsi, Marija Ivanov, Marina Sokovic, Panagiotis Zoumpoulakis

**Affiliations:** 1 Institute of Chemical Biology, National Hellenic Research Foundation, 48, Vas. Constantinou Avenue, 11635 Athens, Greece; ekritsi@eie.gr; 2 Department of Pharmacy, University of Patras, 26504 Patras, Greece; mmatsoukas@cloudpharm.eu; 3 Cloudpharm, Monumental Plaza, Building C, 44, Kifissias Avenue, Marousi, 15125 Athens, Greece; cpotamitis@cloudpharm.eu; 4 School of Chemical Engineering, National Technical University of Athens, 9, Iroon-Polytechneiou-Str, 15773 Athens, Greece; adetsi@chemeng.ntua.gr; 5 Institute for Biological Research “Siniša Stanković”- National Institute of Republic of Serbia, University of Belgrade, 142, Bulevar Despota Stefana, 11000 Belgrade, Serbia; marija.smiljkovic@ibiss.bg.ac.rs (M.I.); mris@ibiss.bg.ac.rs (M.S.)

**Keywords:** *Aspergillus fumigatus*, virtual screening, pharmacophore model, molecular docking, antifungal activity, MIC, MFC, molecular dynamics simulations

## Abstract

The prevalence of invasive fungal infections has been dramatically increased as the size of the immunocompromised population worldwide has grown. *Aspergillus fumigatus* is characterized as one of the most widespread and ubiquitous fungal pathogens. Among antifungal drugs, azoles have been the most widely used category for the treatment of fungal infections. However, increasingly, azole-resistant strains constitute a major problem to be faced. Towards this direction, our study focused on the identification of compounds bearing novel structural motifs which may evolve as a new class of antifungals. To fulfil this scope, a combination of in silico techniques and in vitro assays were implemented. Specifically, a ligand-based pharmacophore model was created and served as a 3D search query to screen the ZINC chemical database. Additionally, molecular docking and molecular dynamics simulations were used to improve the reliability and accuracy of virtual screening results. In total, eight compounds, bearing completely different chemical scaffolds from the commercially available azoles, were proposed and their antifungal activity was evaluated using in vitro assays. Results indicated that all tested compounds exhibit antifungal activity, especially compounds **1**, **2**, and **4**, which presented the most promising minimum inhibitory concentration (MIC) and minimum fungicidal concentration (MFC) values and, therefore, could be subjected to further hit to lead optimization.

## 1. Introduction

In recent decades, fungal infections have been one of the most common and serious health problems worldwide. Their importance is highlighted by the fact that they are responsible for over one million human deaths per year [1]. Additionally, the prevalence of invasive fungal infections has dramatically increased as the size of the immunocompromised population grows. Today more than 5 million fungal species have been identified and, among them, approximately 300 species induce adverse impacts on human life [2].

One of the most common airborne fungal pathogens, causing serious and usually fatal invasive infections, is *Aspergillus fumigatus* [3]. The respiratory tract is the main portal of entry of *Aspergillus fumigatus*, although it can also invades through other parts of the human body [4,5]. The disease caused by fungi of the *Aspergillus* genus is known in the literature as aspergillosis. This disease is generally separated into three categories, based mainly on the range of symptoms it causes, namely allergic bronchopulmonary aspergillosis (ABPA) [6], chronic necrotizing aspergillosis [7], and invasive aspergillosis (IA) [8]. Recent studies proved that IA is responsible for >300,000 cases worldwide [9] and the prevalence of mortality ranges from 30% to 80% [10].

The main attack mechanism against invasive fungal infections is a family of drugs known as antifungals containing four major drug classes: polyenes, echinocandins, flucytosine, and azoles [11]. Amongst the greatest challenges for the design and development of novel antifungals are the decrease of side effects, the increase of efficacy against fungal strains, and the drug resistance of the pathogen species [12,13,14].

Among antifungal agents, the drug class of azoles has been by far the most widely used category in clinical practice [15]. In general, azoles are divided into two main groups, including (i) imidazole and (ii) triazole characteristic rings. In the history of azoles, imidazoles (clotrimazole, miconazole, and ketoconazole) were firstly developed [16]. These drugs exhibit high toxicity, unfavorable side effects, and a plethora of interactions with other drugs, so they were replaced by triazoles. In comparison to imidazoles, the first generation of triazoles, containing itraconazole and fluconazole, possess antifungal activity in a broad range and significantly lower toxicity [11]. However, their clinical usefulness is limited because they are ineffective against some emerging pathogens. The second generation of triazoles (voriconazole, posaconazole, isavuconazole) overcome these limitations and, nowadays, constitute the first-line antifungal agents for treating aspergillosis, displaying lower toxicity and stronger antifungal activity in comparison to other antifungals [17,18].

Azoles inhibit the synthesis of sterols in fungi by inhibiting the cytochrome P450-dependent lanosterol 14-α demethylase (CYP51A) enzyme [19,20]. This enzyme plays a key role in the biosynthesis of ergosterol, an important component of the cell membrane and it catalyzes the demethylation of lanosterol to ergosterol, in a three-step catalysis [21,22]. Particularly, their mechanism is induced through the binding of an unhindered nitrogen atom to the iron heme atom, in the active site of the ERG11 gene that encodes CYP51 enzyme [23]. The crystal structure of CYP51A enzyme of *Aspergillus fumigatus* has not been obtained and their available structural models based on CYP51A of *Homo sapiens* [24] and of *Mycobacterium tuberculosis* [25]. In comparison with lanosterol 14-α demethylase of *Aspergillus fumigatus* the amino acid sequence homology is only 38% and 29%, respectively. The main disadvantage of azoles is the increasing resistance to their activity. This resistance is induced through mutations on the gene that codes the active site of the CYP51A enzyme [26,27,28]. As a result, the scientific community is urged to discover novel non-azole compounds, which could be less prone to resistance.

Thus, the main goal of this study was the discovery of novel scaffolds which could serve as starting structures to develop new drugs with antifungal activity. *Aspergillus fumigatus* was chosen as the target microbe. Towards this aim, a combination of in silico techniques and methodologies were applied, including ligand-based pharmacophore modeling followed by virtual screening of the ZINC database [29]. The screening results were further filtered by the physicochemical properties of azole drugs and the derived hits were subjected to molecular docking studies, an additional method for the final compounds’ selection. Docking scores and the presence of crucial binding interactions to a homology model of the CYP51A enzyme played an essential role in the final compounds’ selection. In total, eight compounds were selected, purchased, and their antifungal activity against different strains of *Aspergillus fumigatus* was evaluated. The results clearly indicate that all examined compounds exhibit antifungal activity and could serve as new starting scaffolds for further hit to lead optimization.

## 2. Results and Discussion

**Figure 1:**
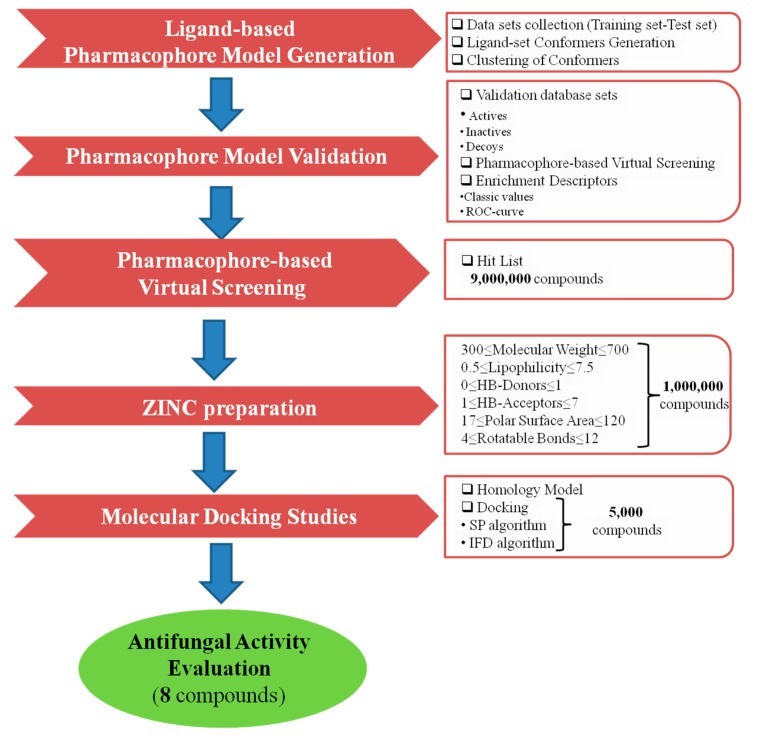
Virtual screening protocol workflow.

The virtual screening process of this study is illustrated in Figure 1.

### 2.1. Ligand-Based Pharmacophore Model Generation

**Figure 2:**
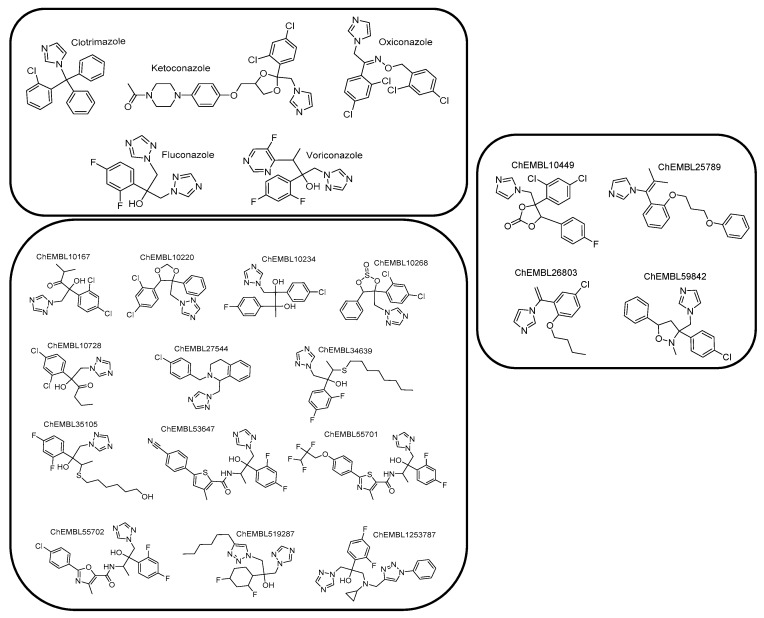
Chemical structures comprising the training set.

**Figure 3:**
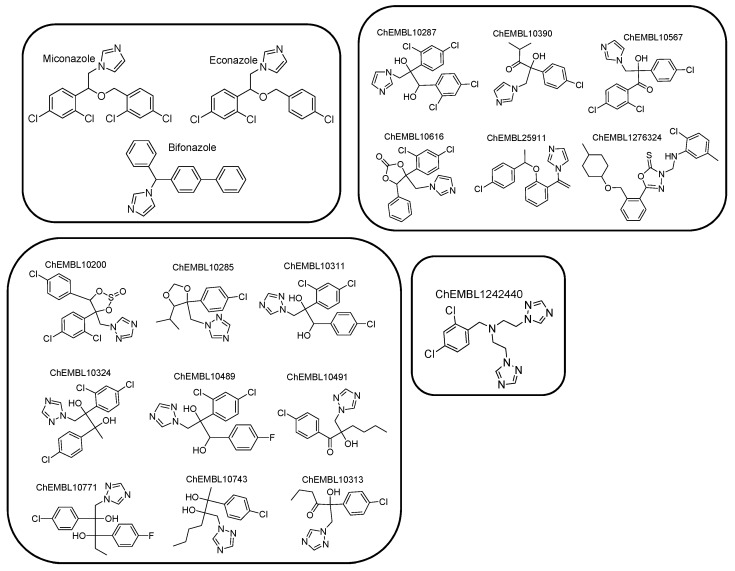
Chemical structures comprising the test set.

Due to the absence of a crystal structure of *Aspergillus fumigatus* CYP51A enzyme, a ligand-based pharmacophore model was generated based on the common features of commercially available active compounds against the tested fungus. Particularly, the training set of 22 active compounds was comprised of five commercially available antifungal drugs from the drug class of azoles (clotrimazole, ketoconazole, oxiconazole, fluconazole, and voriconazole) and 17 active compounds against the tested fungus (1.0 nmol·mL^−1^ < MIC < 305 nmol·mL^−1^), bearing characteristic imidazole and triazole rings (Figure 2, Figure S1, and Table S1 see Supplementary Materials). According to this set, the common chemical features of the compounds were identified, and the pharmacophore model was created. The test set included 20 commercially available active compounds (Figure 3, Figure S2, and Table S2 see Supplementary Materials) against *Aspergillus fumigatus* with structural and functional diversity. More specifically, these compounds belong to two major classes. The first group includes azole drugs (miconazole, econazole, bifonazole) and the second one contains compounds (19 nmol·mL^−1^ < MIC < 350 nmol·mL^−1^) with substituted heterocyclic rings (imidazole, triazole, and tetrazole).

**Figure 4:**
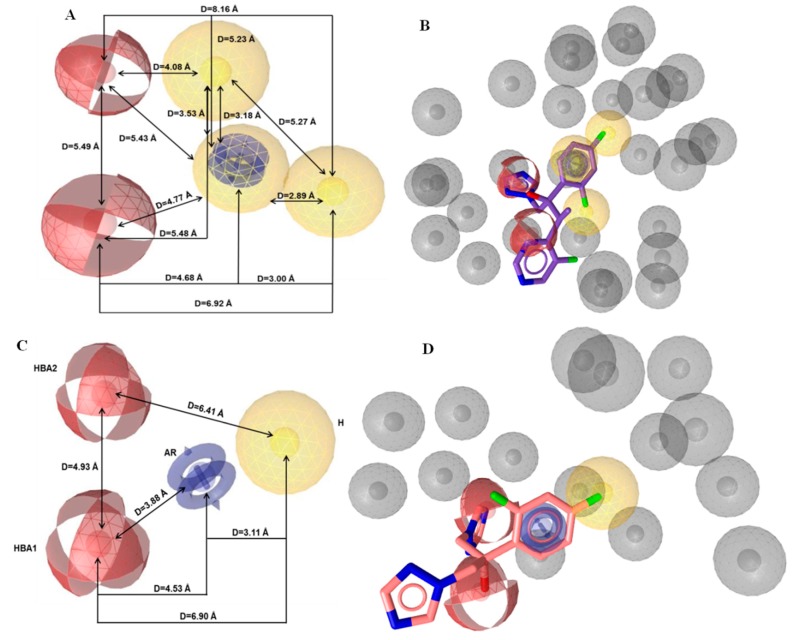
Representation of (**A**) the initial and (**C**) the optimum pharmacophore model features. Fitted of (**B**) voriconazole on the initial pharmacophore features and (**D**) fluconazole on the optimum pharmacophore features. The depiction of the features is colored as follows: hydrogen bond acceptors (HBA) as red spheres, hydrophobic regions (H) as yellow spheres, aromatic rings (AR) as blue rings, and exclusion volumes (Ex. Vol.) as grey spheres. The distances (Å) between the chemical features are illustrated as black lines.

Then, the generation and assessment of a set of 10 pharmacophore hypotheses were carried out. Their examination indicated that the best hypothesis included six common pharmacophore features and all the test set compounds were fitted on them. Particularly, two hydrogen bond acceptors (HBA), three hydrophobic regions (H), one aromatic ring (AR), and 29 exclusion volumes constituted the pharmacophore model (Figure 4A). Among the compounds of the training set, voriconazole fitted optimally (Pharmacophore-Fit Score = 62.23) to the features of the initial model (Figure 4B). Due to the large number of pharmacophore features (6), the present model was considered as improper for practical applications, like virtual screening [30]. Consequently, it was subjected to further optimization based on pharmacophore feature modifications. For this scope, two hydrophobic regions (H) were discarded, while the rest features were preserved in order to keep the major features of azoles, which could play a key role in the binding at the active site of the enzyme. The optimization of the model was completed by changing the size and number (from 29 to 15) of the exclusion volumes to increase the degrees of freedom of the ligands during screening. In Figure 4C,D, the pharmacophore features of the optimized model and the pharmacophore fit of fluconazole (Pharmacophore-Fit Score = 45.21) to the optimum model are illustrated, respectively. In Table 1 the calculated coordinates of the pharmacophore features and the tolerance radii (Å) of the optimum model was presented.

### 2.2. Pharmacophore Model Validation

In general, the reliability of a pharmacophore model depends on the number of biologically active compounds which are retrieved from a structurally diverse compound database and on the number of inactive compounds which are discarded from the same database. For the above scope, the validation database includes three different sets of compounds, including actives, inactives, and decoys. The creation of the decoys set was necessary since the number of available inactive compounds in the literature was inadequate for the study. The enrichment of active compounds is quantified by the hit-rate results from the validation database [31].

**Figure 5:**
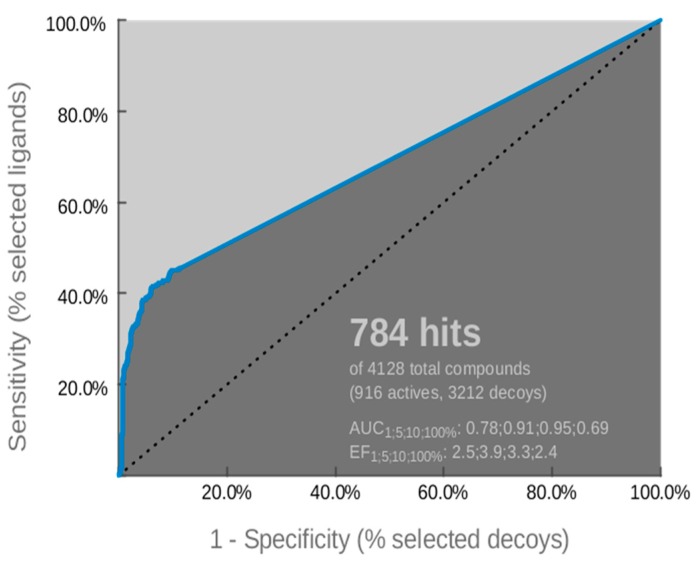
Receiver operating characteristic (ROC) curve of the derived pharmacophore model.

The optimized pharmacophore model was assessed by the receiver operating characteristic (ROC) curve analysis in order to identify its ability to correctly classify a list of compounds as actives or inactives. The quantification of ROC-curve analysis was determined by the area under the curve (AUC) of the examined ROC, as well as by sensitivity (Se), false positive rate (1-Sp), and enrichment factor (EF) values. The ROC-curve is illustrated in Figure 5 and the quantitative key parameters are presented in Table 2. The validation of the model was completed by calculating statistical significance variables (Table 3). The ROC-curve (Figure 5) analysis clearly indicated that the derived model has a good selection score and is certainly better than random selection (AUC= 0.69>0.5). It presents a steep slope at the beginning of screening, indicating a high enrichment of actives among the top-ranked hit list compounds. This fact is amplified by sensitivity (Se) and false positive rate (1-Sp) values (Table 2), as well as the enrichment factor value (EF= 2.4). Additionally, the reliability of the model was confirmed by the fact that it succeeded to retrieve almost 50% of the active compounds and the percentage of retrieved inactives and decoys was by far lower than the actives (Table 3). According to the above results, the generated model was used as a reliable “filter” in virtual screening.

### 2.3. Virtual Screening and Docking Studies

The library “Drugs Now”, a subset of the ZINC database (http://zinc.docking.org/) [29], containing ~11 million compounds which satisfy the drug-likeness criteria [32], was selected for the pharmacophore-based virtual screening [33]. Hits with the top-ranked Pharmacophore-Fit score were subjected to further filtering based on the drug-likeness values of the commercially available antifungal class of azoles (Table S3, see Supplementary Materials). Molecular docking studies were performed on compounds (~1 million) which passed the filtering criteria and showed the highest fit to the pharmacophore model features (Pharmacophore-Fit score range from 41 to 46). Especially, high-throughput docking algorithms were utilized to study the binding mode of the selected compounds to the homology model of *Aspergillus fumigatus* CYP51A enzyme [24]. The lack of the heme ring and any inhibitor from the active site of the homology model was treated by utilizing the respective molecules from the human CYP51A crystal structure (PDB code: 3JUS). This crystal structure includes the heme ring containing a ferric iron atom (Fe^3+^) and the inhibitor R-econazole [34]. The created model was subjected to energy minimization and molecular dynamics simulations to obtain a stable and low energy model.

The final selection of the most promising compounds was based not only on docking scores but also on Pharmacophore-Fit score values and on the visual inspection focusing on the presence of interactions with crucial amino acids. The qualitative criteria of visual inspection were:

(a) The potential metal coordination between the heme ferric iron and the candidate inhibitor [20,28], (distance < 3 Å); and

(b) The formation of π-π interaction with Tyr107 and Tyr121 aminoacids [35].

**Figure 6:**
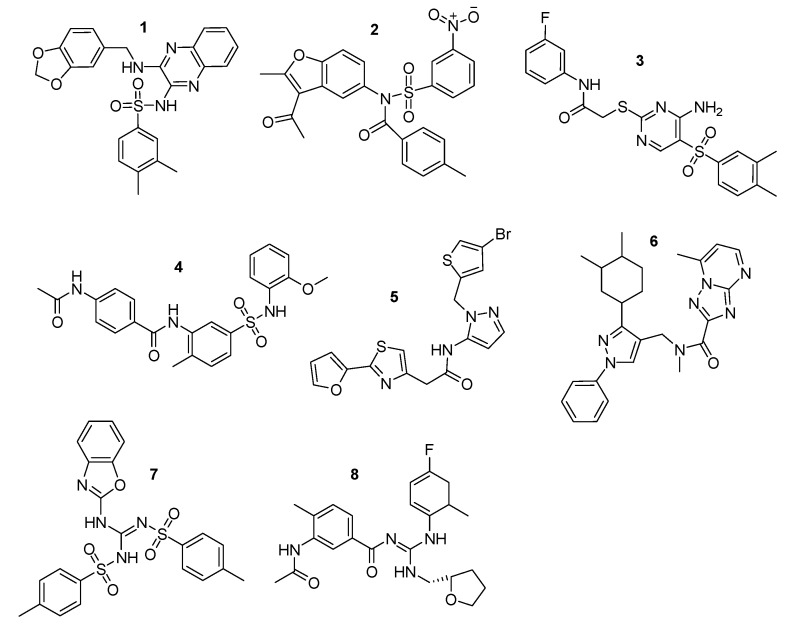
Final compound selection from the pharmacophore-based virtual screening (VS) and the docking studies. Zinc IDs: **1**: ZINC02414861, **2**: ZINC08765786, **3**: ZINC09152123, **4**: ZINC09517045, **5**: ZINC12729365, **6**: ZINC12996228, **7**: ZINC13779062, and **8**: ZINC46132136.

**Figure 7:**
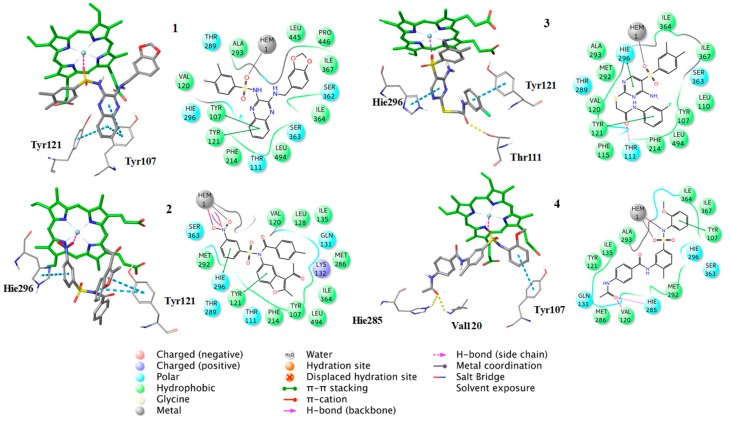
Binding poses of the compounds **1–4**, with the corresponding 2D ligand interaction diagrams, derived from molecular docking studies. Hie is the deprotonated His.

**Figure 8:**
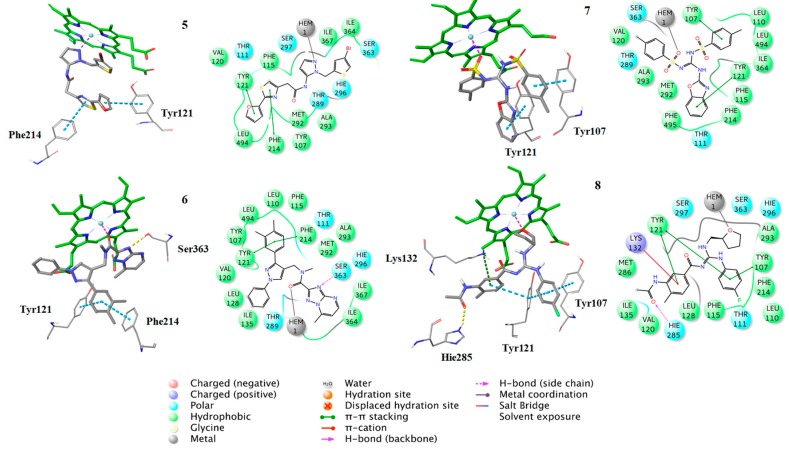
Binding poses of the compounds **5–8**, with the corresponding 2D ligand interaction diagrams, derived from molecular docking studies. Hie is the deprotonated His.

Finally, 8 compounds bearing different chemical scaffolds in comparison to the azole class were selected and acquired for further antifungal activity evaluation (Figure 6). The docking criteria (Glide Score and Emodel), the Pharmacophore-Fit score and the distance from the heme ferric iron of each of the selected compounds are presented in Table 4. The docked poses and the crucial interactions of the above compounds are illustrated in Figure 7 and Figure 8. In Figure S3 (see Supplementary Materials) the pharmacophore-fit of the selected compounds on the features of the optimum model is depicted.

**Figure 9:**
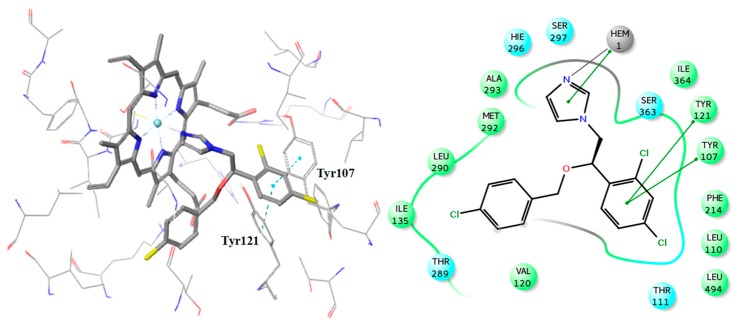
Binding pose of R-econazole with the corresponding 2D ligand interaction diagram.

**Figure 10:**
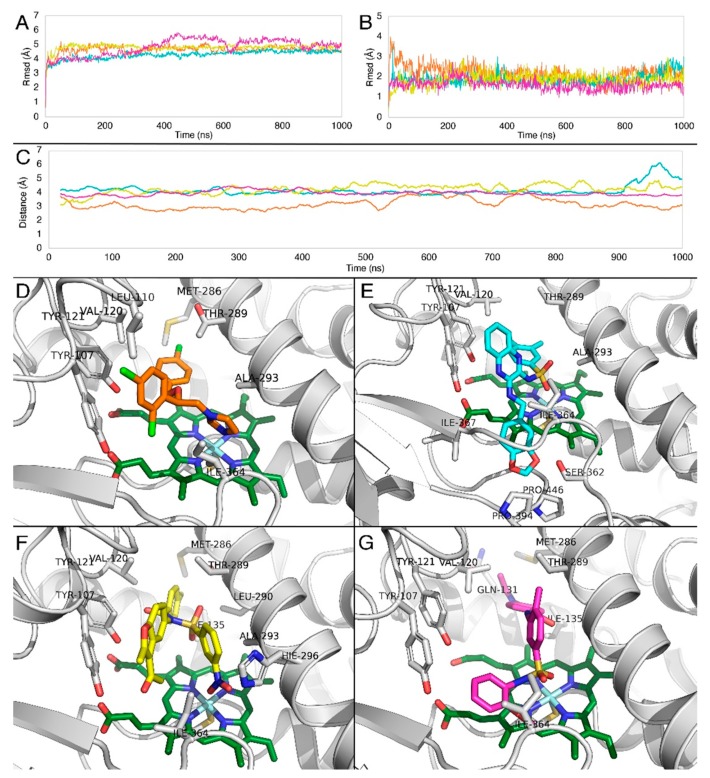
RMSD of (**A**) Cα atoms of CYP51A, (**Β**) ligands along the MD trajectory. Graph colors correspond to the coloring of the four ligands. (**C**) Averaged distances obtained from the molecular dynamics between the heme iron and imidazole nitrogen of R-econazole (orange), center of mass of the two sulfonyl oxygens of compound **1** (cyan), center of mass of the two nitro group oxygens of compound **2** (yellow) and the center of mass of the two sulfonyl oxygens of compound **4** (magenta). Representative complexes obtained from the MD simulations of *Aspergillus fumigatus* CYP51A with (**D**) R-econazole (orange), (**E**) compound **1** (cyan), (**F**) compound **2** (yellow), and (**G**) compound **4** (magenta). The heme molecule is represented in green.

Additionally, the docking analysis suggested that the selected compounds may coordinate the heme metal ion as in the case of azoles binding (Figure 8, Figure 9 and Figure 10). Specifically, compounds **1** (Figure 7) and 7 (Figure 8) develop π-π stacking with the aromatic rings of Tyr107 and Tyr121, as in the case of R-econazole (Figure 9) and both coordinate ferric ion with the sulfonyl group oxygen atom. In the case of compound **2**, the oxygen atom of the nitro group forms a salt bridge with the heme moiety and metal coordination with the iron atom. Moreover, the benzofuran and nitrobenzene rings of the examined compound develop π-π interactions with the side chains of Tyr121 and His296, respectively (Figure 7). The sulfonyl group of compound **3** is responsible, for the formation of metal coordination, as in the case of compound **1** and **7**. Moreover, the halobenzene ring and the pyrimidine ring interact via π-π stacking with the aromatic rings of Tyr121 and His296, respectively. Additionally, its carbonyl group forms a hydrogen bond with the side chain of Thr111 (Figure 7). The oxygen atom of the sulfonyl group of compound **4** develops metal coordination with the heme iron atom, as in the case of compound **1**, **3**, and **7**. Additionally, a salt bridge is created between the N- atom and the heme ring. The methoxy benzene moiety of this compound interacts via a π-π stacking with Tyr107 and its carbonyl group forms two hydrogen bonds with the backbone of Val120 and with the side chain of His285 (Figure 7). The furan and the thiazole ring of compound **5** form π-π interactions with the aromatic rings of Tyr121 and Phe214. The approach towards iron is induced via the nitrogen atom of the imidazole ring of this compound (Figure 8). In the case of compound **6**, the carbonyl group coordinates with the heme iron atom. Furthermore, the o-xylene group forms π-π interactions with Tyr121 and Phe214, as in the case of compound **5**. Additionally, the formation of a hydrogen bond between the nitrogen of triazole ring and the side chain of Ser363 stabilizes the binding (Figure 8). For compound **8**, the oxygen atom of the furan ring is shown to interact with the heme iron atom. Its halobenzene ring makes π-π stacking with the aromatic rings of Tyr107 and Tyr121. Additionally, the methylbenzene group forms a π-π interaction with Tyr121. Moreover, a hydrogen bond is formed with the side chain of His285 and a π-cation interaction is presented with Lys132 (Figure 8). The interactions of the selected compounds in the active site of *Aspergillus fumigatus* CYP51A modeled enzyme, are presented in Table 5.

### 2.4. Antifungal Activity Evaluation

In vitro assays were performed to determine the antifungal activity of the selected compounds, against different strains of *Aspergillus fumigatus*. The minimal inhibitory concentrations (MIC) and minimal fungicidal concentrations (MFC) were identified for the selected compounds compared to reference azole drugs, econazole, and ketoconazole (Table 6).

The in vitro results indicated that all tested compounds displayed antifungal activity against the two strains of *Aspergillus fumigatus*. Especially, in the case of the clinical isolate the MIC and MFC values ranging from 0.12 to 0.27 μmol·mL^−1^ and from 0.40 to 0.54 μmol·mL^−1^, respectively. Additionally, the observed MIC (0.05–0.26 μmol·mL^−1^) and MFC (0.10–0.54 μmol·mL^−1^) values of the tested compounds against the ATCC204305 strain possessed higher antifungal activity compared to clinical isolate strain. It is obvious that compounds **1** and **2** presented the highest antifungal activity against the ATCC204305 strain, whereas compound **4** achieved the highest inhibitory effect in the case of clinical isolate *Aspergillus fumigatus*. It should be mentioned that compound **7** exhibited the lowest antifungal activity against both examined strains.

A general observation is that the MIC and MFC values of the hit compounds were remarkably different from those of econazole compared to the predictions of the molecular docking. Since the in vitro testing of compounds was performed directly to fungal strains (and not to CYP51 protein determining binding affinities) there may be several reasons explaining the different activities. These may include fungal cell wall permeability, cell membrane permeability, solubility of tested compounds, etc. Indeed, Table S4 presents the predicted values of physicochemical parameters such as lipophilicity and polar surface area. From these results we may notice that econazole is more lipophilic with a significantly lower polar surface area (27.05) compared to tested compounds (109.95 for compound **4** to 136.18 for compound **2**) which may constitute a putative explanation.

### 2.5. Molecular Dynamics Simulations

In order to evaluate the stability of compounds **1**, **2**, and **4**, compared to R-econazole, the docking poses of the four molecules were subjected to unconstrained molecular dynamics simulations for 1 μs each. In all cases, the RMSD of the structural model backbone was reasonable (Figure 10A), as well as the ligand RMSD (Figure 10B).

To further evaluate the interactions which contribute to complex stability, the averaged distances were measured from the molecular dynamics simulations between the heme iron and the center of mass of the two sulfonyl oxygens of compound **1** (cyan), the center of mass of the two nitro group oxygens of compound **2** (yellow),the center of mass of the two sulfonyl oxygens of compound **4** (magenta), and the imidazole nitrogen of R-econazole (orange).

Results indicate (Figure 10C) that the imidazole nitrogen of R-econazole, the sulfonyl group oxygens of compounds **1** and **4** and nitro group oxygens of compound **2** were found in constant proximity to Fe^3+^. Although these interactions with heme iron remain stable for all tested compounds as shown in the distance profiles along their trajectories (Figure 10C), we postulate that the forces maintaining such a stable binding profile are hydrophobic. This could be explained by the fact that the RMSD of these three compounds was stable along the trajectories as shown in Figure 10B.

Moreover, the three compounds under investigation share common binding characteristics. R-econazole forms steady hydrophobic contacts between the two-chlorine bearing aromatic rings with mainly Thr289, Leu110, and Val120 and the imidazole ring is stabilized by Ala293 (Figure 10D). Compound **1**, as a larger in size compound, besides hydrophobic contacts between its aromatic groups and mainly Thr289, Leu110, and Val120, bears an extra benzo-dioxole group, which penetrates deeper to interact with the two prolines Pro394 and Pro446, as well as Ile364 and Ser362 (Figure 10E). Compound **2** forms similar hydrophobic interactions with Val120, Tyr121, as well as Tyr107. The benzyl group attached to the Fe interacting nitro group is also stabilized by Ala239 (Figure 10F). Compound **4** has a similar interaction profile, forming aromatic-hydrophobic interactions with Thr289, Val120, and Ile364, and hydrophobic interactions with Met286 (Figure 10G).

## 3. Materials and Methods

### 3.1. Pharmacophore Model Generation

For the creation and the validation of the pharmacophore model, LigandScout 4.0 Advanced software was used (InteLigand, GmbH, Vienna, Austria [36,37]. Due to the absence of a crystal structure of fungi CYP51A, a ligand-based pharmacophore approach was applied. The compounds with known biological activity against *Aspergillusfumigatus* were extracted from the ChEMBL database [38] and were utilized for the creation of the training and the test set. The training set included five representative drugs from the class of azoles and 17 active compounds (1 nmol·mL^−1^≤ MIC ≤ 310 nmol·mL^−1^) with chemical diversity (Figure 2). According to this set, the common chemical features of the compounds were aligned, and the model was created. The test set contained 3 commercially available antifungal drugs (miconazole, econazole, and bifonazole) and 19 active compounds (19 nmol·mL^−1^ ≤ MIC ≤ 350 nmol·mL^−1^) (Figure 3). All compounds were converted from 2D to 3D and then were minimized in Maestro 2013 (Schrodinger, LLC, New York, NY, USA) software. Training set compounds were prepared in pH 8.0 ± 0.5 using the LigPrep module of MAESTRO software (Schrodinger, LLC). In the case of the test set, all compounds were prepared to retain their chiralities. The chemical structures and activities of training and test sets are presented in Tables S1 and S2 (see Supplementary Materials), respectively. The OMEGA algorithm was applied for the generation of conformations of the ligand set [39] and the maximum number of conformations per ligand was set to 50. Then, the ligand set was clustered according to the pharmacophore alignment score and 10 pharmacophore hypotheses were created. The performed scoring function was the “Pharmacophore-Fit and Atom Overlap”, the selected pharmacophore type was the “merged” pharmacophore features, and the number of omitted pharmacophore features was 6 (Figure 4).

### 3.2. Evaluation of Validation Results

For the validation process, three libraries were created by using the ChEMBL [38] database. The first one included 916 active compounds, the second, 117 inactive compounds and, due to the small number of inactive compounds available in literature, adecoy library was also created, consisting of 3095 compounds structurally similar to the active ones, but experimentally not tested for biological activity. The creation of this library was based on the application of an in-house protocol.

In this work, the reliability and accuracy of the generated model was evaluated by using classic enrichment parameters, as sensitivity (Se) (Equation (1)), specificity (Sp) (Equation (2)), enrichment factor (EF) (Equation (3)), and the receiver operating characteristic (ROC) curve [40].

Sensitivity (Se) determines the percentage of truly active compounds selected during virtual screening and it is defined as:

Se = Selected Actives/All Actives(1)

Specificity (Sp) describes the percentage of truly inactive compounds rejected during virtual screening and it is defined as:

Sp = Discarded (Inactives + Decoys)/All (Inactives + Decoys)(2)

Enrichment factor measures how much better the method is than a random selection by considering the fraction of found actives compounds in the top of the ranked hit list, compared to the ration of compounds in the entire database:

EF = [(Hits top selected/Compounds top selected)]/[(Total Hits/All Compounds)](3)

The ROC-curve is a graphical plot of Se versus 1-Sp and the area under the curve (AUC) is measured by:

(4) $$\mathrm{AUC}\;=\;\sum_{x=2}^{n}Se\left(x\right)[\left(1-Sp\right)\left(x\right)-\left(1-Sp\right)\left(x-1\right)$$

### 3.3. Pharmacophore-Based Virtual Screening

Pharmacophore-based virtual screening was applied to the ZINC database (http://zinc.docking.org/) subset “Drugs Now”, which includes approximately 11 million compounds. The idbgen-tool of LigandScout [36,37] was used to convert this pool of compounds to the appropriate screening format. Twenty-five conformers per compound were created by the OMEGA algorithm [39].

The higher pharmacophore-fit score values (43–46) were utilized as the main filter of the hit list. The next filter was based on the values of the drug-likeness criteria of the azole drug class (molecular weight, lipophilicity, number of hydrogen-bond donors, number of hydrogen-bond acceptors, polar surface area and number of rotatable bonds) (Table S3, see Supplementary Materials). All drugs were prepared in the optimum pH = 8.0 ± 0.5 by the LigPrep [41] version 2.7 (Schrodinger, LLC) module and their physicochemical properties were calculated by the QikProp version 3.9 module of MAESTRO (Schrodinger, LLC). One out of nine million compounds which were screened fit to the pharmacophore model features and satisfy the drug-likeness criteria of azoles.

### 3.4. Molecular Docking Studies

The derived compounds were subjected to further filtering through molecular docking studies at the binding site of the *Aspergillus fumigatus* CYP51A constructed model [24]. This model was based on the human CYP51A model ortholog, providing a high-quality model. The heme ring and the inhibitor econazole (R) were transferred from the human crystal structure (PDB: 3JUS) and were aligned to the constructed model. The hits were docked with the Standard Precision mode of Glide, version 6.0 (Schrodinger, LLC) [42,43]. The resulting poses (5000 compounds) were visually inspected and the most promising ones were further docked with the Induced-Fit protocol 2013-2, Glide, version 5.9, Prime, version 3.2 (Schrodinger, LLC) [44].

### 3.5. Antifungal ActivityEvaluation

For the antifungal bioassays, two strains of *Aspergillus fumigatus* were used: *Aspergillus fumigatus* (ATCC 204305), and *Aspergillus fumigatus* (human clinical isolate). The organisms are deposited at the Mycological Laboratory, Department of Plant Physiology, Institute for Biological Research “Siniša Stankovic,” Belgrade, Serbia.

The micromycetes were maintained on malt agar and the cultures were stored at 4°C and sub-cultured once a month. The antifungal assay was carried out by a modified microdilution technique [45,46] in order to determine the minimum inhibitory (MIC) and minimum fungicidal concentrations (MFC) of the examined compounds. Briefly, the fungal spores were washed from the surface of agar plates with sterile 0.85% saline containing 0.1% Tween 80 (*v/v*). The spore suspension was adjusted with sterile saline to a concentration of approximately 1.0×10^5^ in a final volume of 100 μL per well. The examined compounds were dissolved in 5% of DMSO, serially diluted in broth malt medium after which fungal inoculum was added. The microplates were incubated for 72 h at 28°C. The lowest concentrations without visible growth (in a binocular microscope) were defined as MICs. The fungicidal concentrations (MFCs) were determined by serial sub cultivation of 2 μL of the wells content into microtiter plates containing 100 μL of broth per well and further incubation for 72 h at 28°C. The lowest concentration with no visible growth was defined as MFC indicating 99.5% killing of the original inoculum. The commercial antifungals econazole and ketoconazole were used as positive controls.

### 3.6. Molecular Dynamics

All MD simulations were performed using GROMACS 2018 v3 (University of Groningen& Royal Institute of Technology, Uppsala University, Groningen, Netherlands) [47]. The structural model, described by Fraczeket al. [24] in complex with heme (covalently) and compounds R-econazole, compound **1**, compound **2**, and compound **4**, in each case as a result of the docking previously described, was inserted in a pre-equilibrated box containing water and a 0.15M concentration of Na and Cl ions. The latest AMBER99SB-ILDN [48] force field was used for all the dynamics simulations along with the TIP3P water model. Force field parameters for the ligands were generated using the general Amber force field (GAFF) and HF/6-31G*-derived RESP atomic charges [49]. AMBER-compatible heme parameters were derived from elsewhere [50]. Each system consisted of the protein, the ligand, ~15,000water molecules, and ~160 ions in a 10 × 10 × 10nm simulation box. The model systems were energy minimized and subsequently subjected to a 20 ns MD equilibration, with positional restraints on protein Cα atoms. These restraints were released and 1000 ns MD trajectories were produced at a constant temperature of 300K using separate v-rescale thermostats for the protein, the ligand, and solvent molecules. A time step of 2 fs was used, and all bonds were constrained using the LINCS algorithm. Lennard–Jones interactions were computed using a cutoff of 10 Å, and the electrostatic interactions were treated using PME with the same real-space cutoff as described before [51].

## 4. Conclusions

In the present study, a combination of in silico techniques and methodologies were implemented to identify novel scaffolds with antifungal activity against *Aspergillus fumigatus* targeting the fungal cytochrome P450 dependent lanosterol 14-α demethylase (CYP51A) enzyme. For this scope, a carefully designed and validated ligand-based pharmacophore model was used to screen the ZINC database. Compounds that passed the pharmacophore filtering were further examined using molecular docking studies. In total, eight compounds were selected, six of which have different azole structural motifs. The MIC and MFC values from the antifungal activity tests showed that all compounds present antifungal activity in comparison to commercially available antifungals and could serve as starting materials for further modifications. Especially, compounds **1**, **2**, and **4** exhibited the highest activity and were further studied to explore their mechanism of action on the CYP51A enzyme by means of molecular dynamics simulations. All three compounds were found to share common binding characteristics. Importantly, the imidazole nitrogen of R-econazole, the sulfonyl group oxygens of compounds **1** and **4**, and nitro group oxygens of compound **2**, were found in constant proximity to Fe^3+^. The role of hydrophobic interactions with amino acids Thr289, Leu110, Val120, Tyr107, and Tyr121to the stability of the complexes was also observed.

In conclusion, compounds **1**, **2**, and **4** are the most promising scaffolds that differentiate from classical azoles and could serve as starting points for further hit-to-lead optimization.

## Figures and Tables

**Table 1:** Pharmacophore features coordinates and tolerance radii (Å) of the optimized pharmacophore model.

		Cartesian Coordinates		
Feature Type	Radius (Å)	X	Υ	Z
HBA1	1.65	1.64	0.02	1.77
HBA2	1.65	−0.17	−3.17	−1.52
AR	1.05	4.07	−2.27	−0.20
H	1.65	6.07	−4.53	−0.93

**Table 2:** ROC performance of the examined pharmacophore model.

Sensitivity (Se)	0.45
False Positive Rate (1-Sp)	0.06
Enrichment Factor (EF)	2.4
Area Under Curve (AUC)	0.69

**Table 3:** Values of the calculated statistical significance variables.

Variable	Value	Variable	Value
A	916	D_t_	4128
I	117	H_t_	784
D	3095	H_a_	417
%A	45.52	H_i_	7
%I	5.98	H_d_	360
%D	11.63		

The number of (A): actives, (I): inactives and (D): decoys, in the database. (D_t_) the total number of compounds in the database and (H_t_) the number of hits retrieved. (H_a_), (H_i_), and (H_d_) the number of actives, inactives and decoys in the hit list, respectively. The percentage of known i) actives (%A), ii) inactives (%I), and iii) decoys (%D) in the hit list.

**Table 4:** Docking Score and Emodel energy contributions, Pharmacophore-Fit score values, and distance from the heme ferric iron atom of the selected compounds and the known inhibitor R-econazole.

Compound	Docking Score (kcal·mol^−1^)	Emodel (kcal·mol^−1^)	Pharmacophore-Fit Score	Distance Fe^3+^(Å)
**1**	−8.98	−91.44	45.14	2.49
**2**	−7.85	−88.15	44.77	2.40
**3**	−8.37	−91.08	43.41	2.36
**4**	−7.55	−93.28	45.13	2.21
**5**	−7.88	−80.73	43.28	2.45
**6**	−10.32	−119.77	43.62	2.29
**7**	−9.80	−81.13	44.28	2.48
**8**	−8.42	−79.25	44.97	2.35
**R-econazole**	−7.56	−73.64	45.03	2.56

**Table 5:** Interactions of compounds **1–8**, developed in the active site of *Aspergillus fumigatus* CYP51A modeled enzyme.

Compound	Interactions				
**1**	MC	π-π Tyr107	π-π Tyr121	-	
**2**	MC	-	π-π Tyr121	π-π His296	
**3**	MC	-	π-π Tyr121	π-π His296	HB Thr11
**4**	MC	π-π Tyr107	-	HB His285	HB Val120
**5**	MC	-	π-π Tyr121	π-π Phe214	
**6**	MC	-	π-π Tyr121	π-π Phe214	HB Ser363
**7**	MC	π-π Tyr107	π-π Tyr121	-	
**8**	MC	π-π Tyr107	π-π Tyr121	HB Hie285	π-c Lys132
**R-econazole**	MC	π-π Tyr107	π-π Tyr121	π-π heme	

MC: Metal Coordination.

**Table 6:** Minimal inhibitory (MIC) and fungicidal (MFC) concentration values of the proposed (**1**–**8**) compounds (μmol·mL^−1^).

	Aspergillus fumigatus Clinical Isolate		Aspergillus fumigatus ATCC 204305	
Compound	MIC	MFC	MIC	MFC
**1**	0.2024 ± 0.001^d^	0.4047 ± 0.002^c^	0.1012 ± 0.001^d^	0.4047 ± 0.003^d^
**2**	0.2028 ± 0.001^d^	0.4056 ± 0.002^c^	0.0507 ± 0.0008^c^	0.1014 ± 0.001^c^
**3**	0.2096 ± 0.001^d^	0.4192 ± 0.003^c^	0.2096 ± 0.001^e^	0.4192 ± 0.002^d^
**4**	0.1190 ± 0.001^c^	0.4762 ± 0.003^d^	0.2381 ± 0.001^e^	0.4762 ± 0.002^e^
**5**	0.2083 ± 0.001^d^	0.4166 ± 0.002^c^	0.2083 ± 0.001^e^	0.4166 ± 0.002^d^
**6**	0.2053 ± 0.001^d^	0.4106 ± 0.002^c^	0.2053 ± 0.002^e^	0.4106 ± 0.003^d^
**7**	0.2693 ± 0.001^e^	0.5386 ± 0.004^d^	0.2693 ± 0.001^f^	0.5386 ± 0.004^e^
**8**	0.1986 ± 0.001^d^	0.3972 ± 0.002^c^	0.1986 ± 0.001^e^	0.3972 ± 0.002^d^
**Ketoconazole**	0.0023 ± 0.00002^b^	0.0046 ± 0.00004^b^	0.0023 ± 0.00001^b^	0.0046 ± 0.00004^b^
**Econazole**	0.1754 × 10^−3^ ± 0.01 ×10^−3a^	0.3508 × 10^−3^ ± 0.02 × 10^−3a^	0.0877 × 10^−3^ ± 0.002 × 10^−3a^	0.1754 × 10^−3^ ± 0.002 × 10^−3a^

Experiments were performed in duplicate and repeated three times. Values are expressed as means ± SD. In each column, different letters mean significant differences between samples (*p* < 0.05).

## Supplementary Materials

The following are available online. Table S1. MIC values (nmol·mL^−1^) of training set compounds; Table S2. MIC values (nmol·mL^−1^) of test set compounds; Table S3. The values of the drug-likeness criteria of the azole drug class.
