# Supplementary material for: Novel Hit Compounds as Putative Antifungals: The Case of Aspergillus fumigatus
Source: Molecules. 2019 Oct 25;24(21):3853. doi: 10.3390/molecules24213853 (PMC6864791; doi:10.3390/molecules24213853)
Supplement: Supplementary file 1 [file molecules-24-03853-s001.zip › supplementary-final/supplementary-final - checked.pdf]

Supplementary Materials

Table S1: MIC values (nmol·mL^{-1}) of training set compounds

**Compound**	**MIC (nmol·mL^{-1})**	**Compound**	**MIC (nmol·mL^{-1})**
Clotrimazole	2.9-36.25	ChEMBL10449	122.78
Ketoconazole	0.235-380	ChEMBL25789	17.79
Oxiconazole	4.66-14.56	ChEMBL26803	1.45
Fluconazole	20.41	ChEMBL59842	197.83
Voriconazole	0.258-22.90	ChEMBL34639	0.98
ChEMBL10167	304.70	ChEMBL35105	2.02
ChEMBL10220	33.22	ChEMBL53647	18.03
ChEMBL10234	276.40	ChEMBL55701	3.67
ChEMBL10268	121.87	ChEMBL55702	81.99
ChEMBL10728	152.35	ChEMBL519287	17.67
ChEMBL27544	11.81	ChEMBL1253787	69.94

Table S2: MIC values (nmol·mL^{-1}) of test set compounds

**Compound**	**MIC (nmol·mL^{-1})**	**Compound**	**MIC (nmol·mL^{-1})**
Miconazole	0.77-1.2	ChEMBL10285	324.91
Econazole	8.12	ChEMBL10311	62.71
Bifonazole	480.00	ChEMBL10313	42.55
ChEMBL10287	231.41	ChEMBL10324	60-120
ChEMBL10390	85.39	ChEMBL10489	65.41
ChEMBL10567	63.18	ChEMBL10491	321.91
ChEMBL10616	51.38	ChEMBL10743	154.41
ChEMBL25911	19.09	ChEMBL10771	66.52
ChEMBL1276324	55.31	ChEMBL1242440	349.49
ChEMBL10200	56.215	ChEMBL152027	2.264

Table S3: Values of the druglikeness criteria of Azole drug class

Compound	Molecular Weight (MW)	Lipophilicity (LogP)	HB-Donor (HBD)	HB-acceptor (HBA)	Polar Surface Area (PSA)	Rotatable Bonds (RB)
**Albaconazole**	431.82	2.82	1	5	85.83	5
**Bifonazole**	310.39	5.23	0	1	17.82	4
**Cyproconazole**	291.77	2.85	1	3	50.94	5
**Econazole**	381.68	5.35	0	2	27.05	6
**Fluconazole**	306.27	0.56	1	5	81.65	5
**Itraconazole**	705.63	7.31	0	7	104.7	11
**Ketoconazole**	531.43	4.19	0	5	69.06	7
**Miconazole**	416.12	5.96	0	2	27.05	6
**Posaconazole**	700.77	5.41	1	7	115.7	12
**Propiconazole**	342.22	4.33	0	4	49.17	5
**Ravuconazole**	437.46	4.14	1	5	115.86	6
**Tebuconazole**	307.81	3.69	1	3	50.94	6
**Tioconazole**	387.71	5.3	0	2	55.29	6
**Voriconazole**	349.31	1.82	1	5	76.72	5

Table S4: Values of the druglikeness criteria of selected compounds 1-8

Compound	Molecular Weight (MW)	Lipophilicity (LogP)	HB-Donor (HBD)	HB-acceptor (HBA)	Polar Surface Area (PSA)	Rotatable Bonds (RB)
**1**	462.52	4.99	2	6	110.82	6
**2**	492.50	4.45	0	5	136.18	6
**3**	446.52	3.83	2	5	148.72	6
**4**	452.40	3.31	2	5	109.95	7
**5**	448.34	3.62	0	4	117.40	6
**6**	451.52	3.83	0	5	81.73	5
**7**	484.55	4.50	0	7	147.49	7
**8**	412.46	2.83	1	4	91.82	7

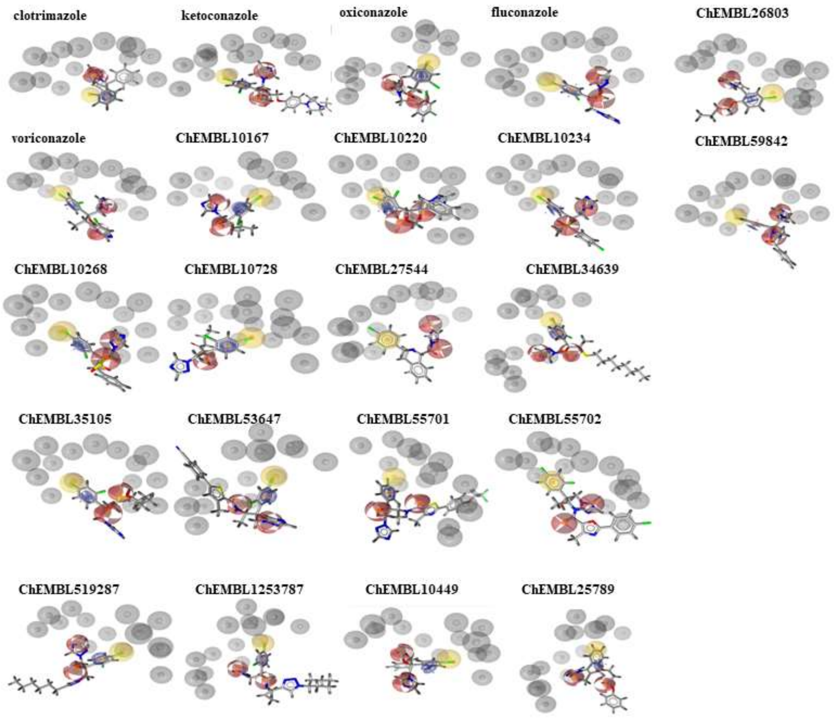

Figure S1: The fit of the training set compounds on the optimum pharmacophore model features. The depiction of the features is colored as follows: hydrogen bond acceptors (HBA) as red spheres, hydrophobic regions (H) as yellow spheres, aromatic rings (AR) as blue rings and exclusion volumes (Ex. Vol.) as grey spheres.

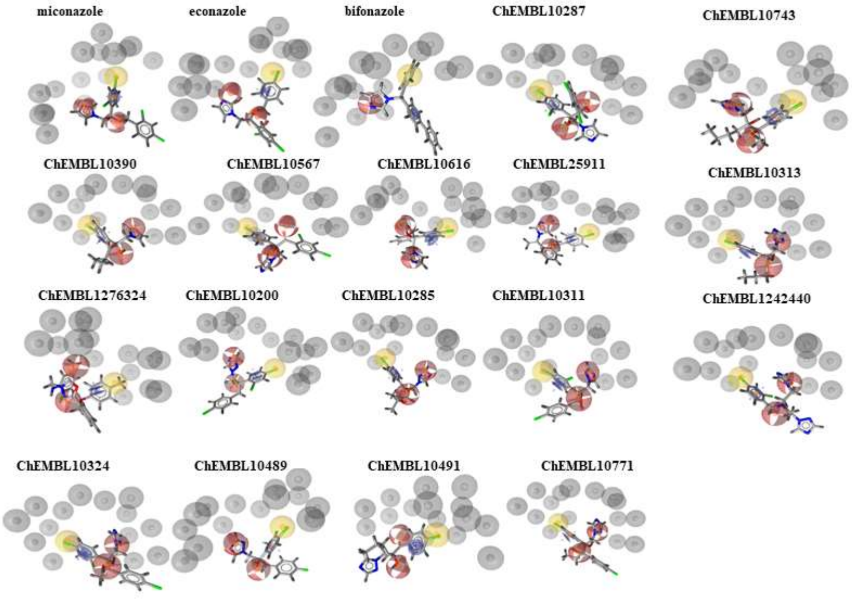

Figure S2: The fit of the test set compounds on the optimum pharmacophore model features. The depiction of the features is colored as follows: hydrogen bond acceptors (HBA) as red spheres, hydrophobic regions (H) as yellow spheres, aromatic rings (AR) as blue rings and exclusion volumes (Ex. Vol.) as grey spheres.

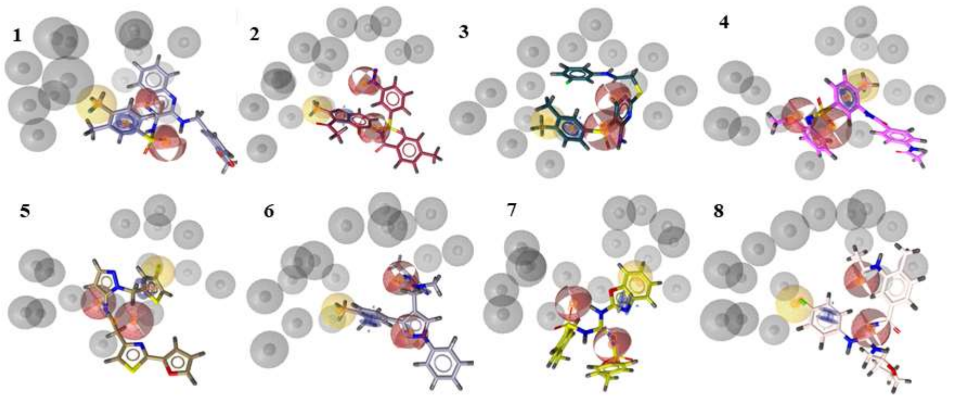

Figure S3: The pharmacophore-fit of the final compounds on the features of the optimum model. The depiction of the features is colored as follows: hydrogen bond acceptors (HBA) as red spheres, hydrophobic regions (H) as yellow spheres, aromatic rings (AR) as blue rings and exclusion volumes (Ex. Vol.) as grey spheres.
