# Supplementary material for: Novel Hit Compounds as Putative Antifungals: The Case of Aspergillus fumigatus
Source: Molecules. 2019 Oct 25;24(21):3853. doi: 10.3390/molecules24213853 (PMC6864791; doi:10.3390/molecules24213853)
Supplement: Supplementary file 1 [file molecules-24-03853-s001.zip › supplementary-final/Supplementary Figures.zip!/Figure S3.pdf]

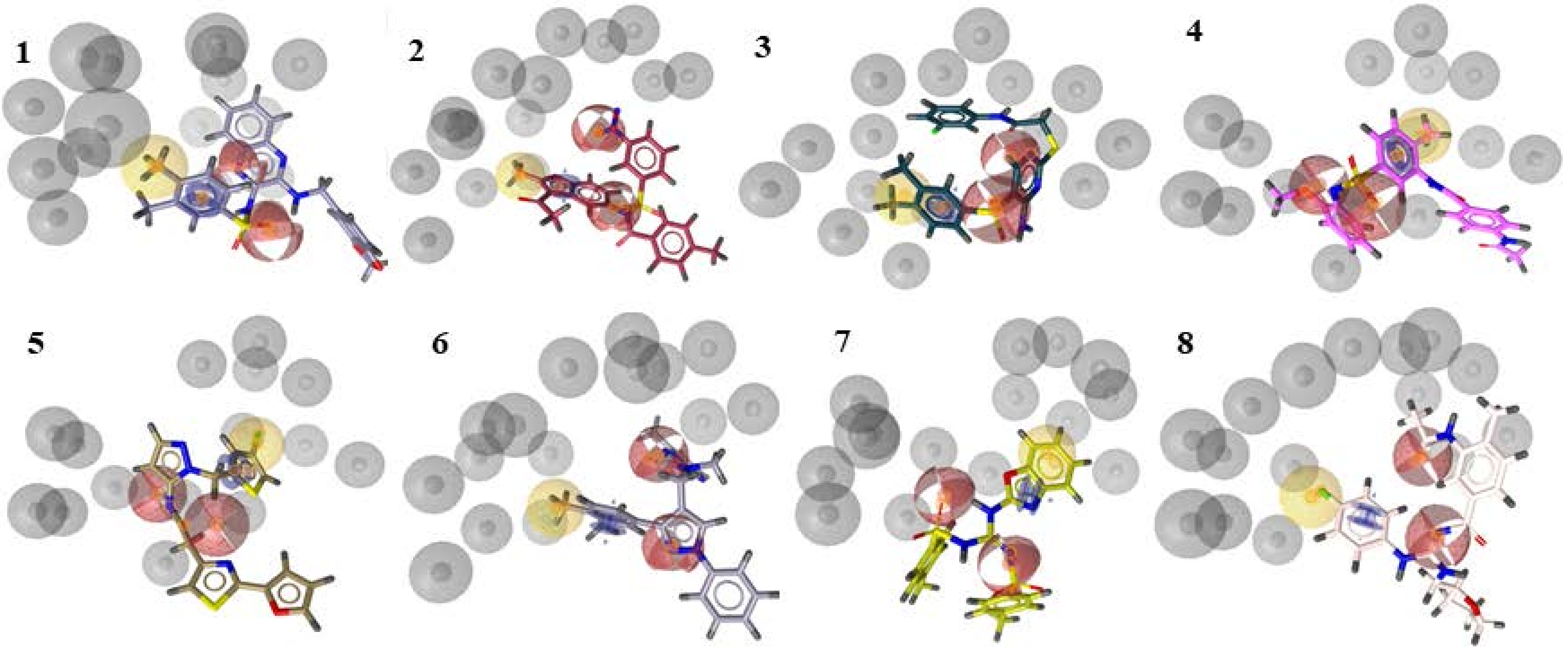
1
2
3
4
5
6
7
8
