# Supplementary material for: Novel Hit Compounds as Putative Antifungals: The Case of Aspergillus fumigatus
Source: Molecules. 2019 Oct 25;24(21):3853. doi: 10.3390/molecules24213853 (PMC6864791; doi:10.3390/molecules24213853)

clotrimazole

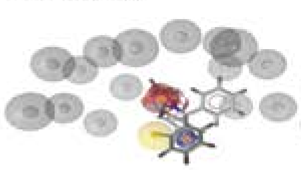

ketoconazole

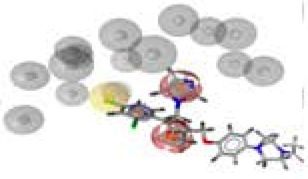

oxiconazole

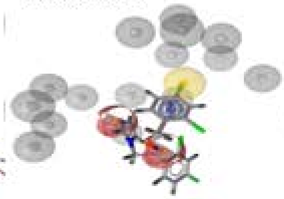

fluconazole

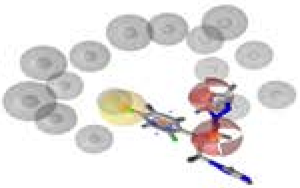

ChEMBL26803

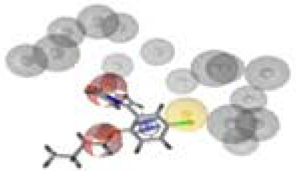

voriconazole

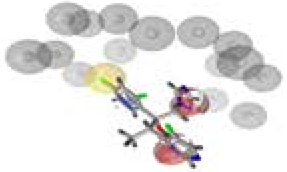

ChEMBL10167

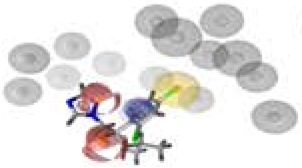

ChEMBL10220

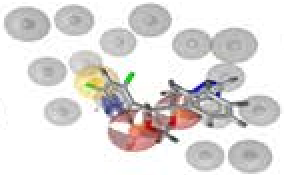

ChEMBL10234

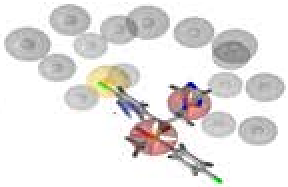

ChEMBL59842

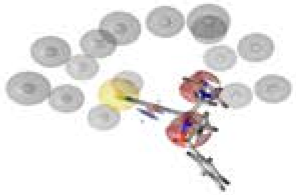

ChEMBL10268

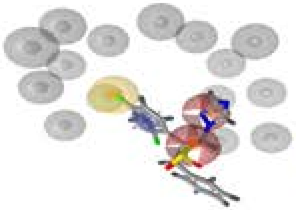

ChEMBL10728

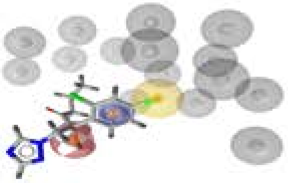

ChEMBL27544

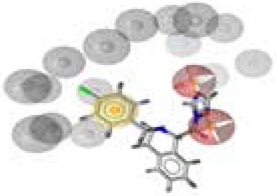

ChEMBL34639

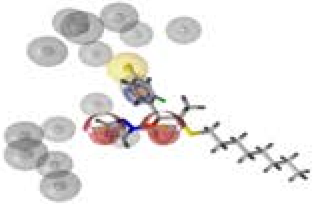

ChEMBL35105

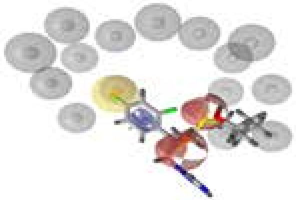

ChEMBL53647

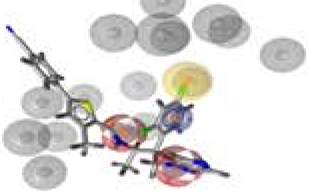

ChEMBL55701

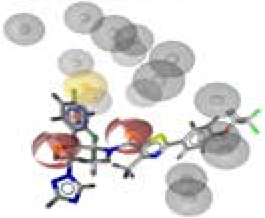

ChEMBL55702

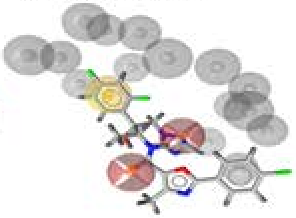

ChEMBL519287

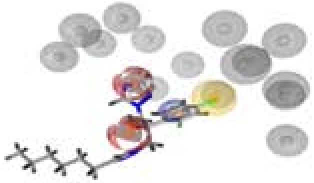

ChEMBL1253787

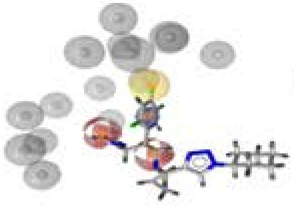

ChEMBL10449

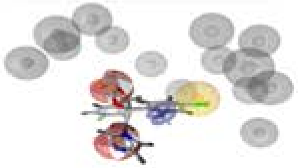

ChEMBL25789

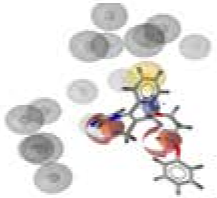

Supplement: Supplementary file 1 [file molecules-24-03853-s001.zip › supplementary-final/Supplementary Figures.zip!/Figure S1.pdf]
