# Supplementary material for: Novel Hit Compounds as Putative Antifungals: The Case of Aspergillus fumigatus
Source: Molecules. 2019 Oct 25;24(21):3853. doi: 10.3390/molecules24213853 (PMC6864791; doi:10.3390/molecules24213853)

miconazole

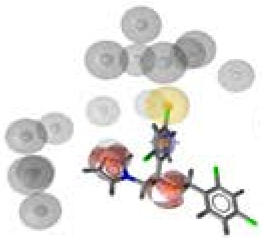

econazole

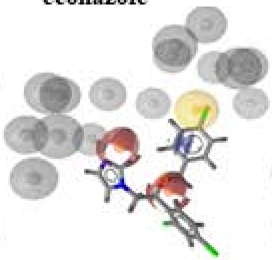

bifonazole

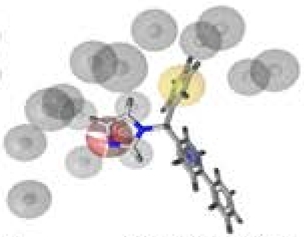

ChEMBL10287

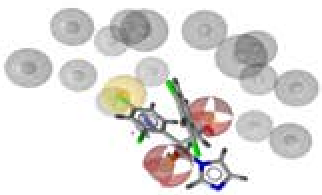

ChEMBL10743

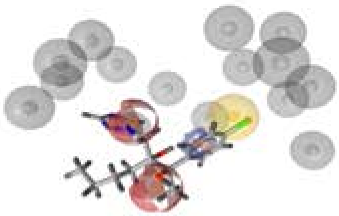

ChEMBL10390

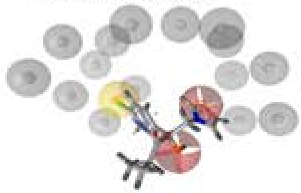

ChEMBL10567

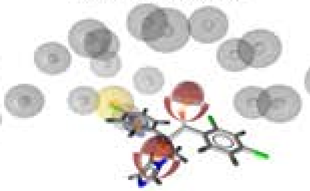

ChEMBL10616

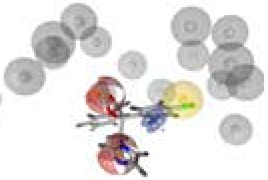

ChEMBL25911

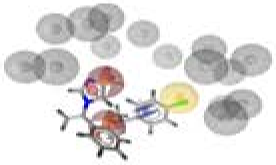

ChEMBL10313

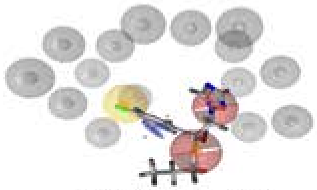

ChEMBL1276324

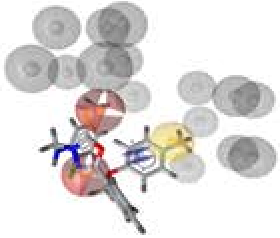

ChEMBL10200

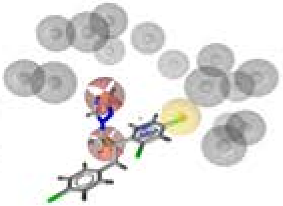

ChEMBL10285

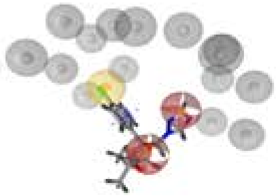

ChEMBL10311

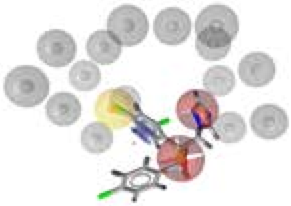

ChEMBL1242440

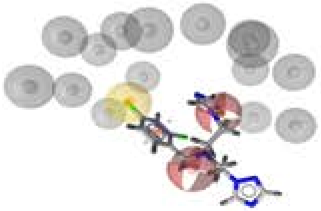

ChEMBL10324

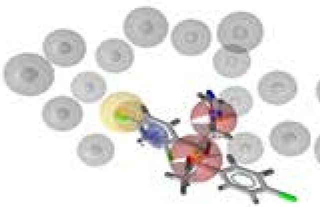

ChEMBL10489

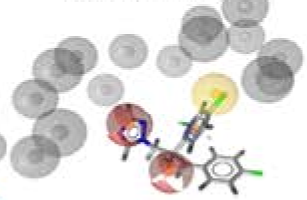

ChEMBL10491

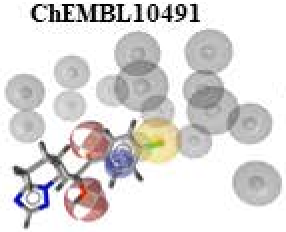

ChEMBL10771

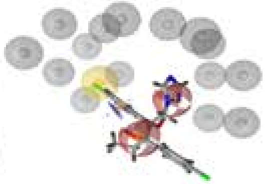

Supplement: Supplementary file 1 [file molecules-24-03853-s001.zip › supplementary-final/Supplementary Figures.zip!/Figure S2.pdf]
